# Supplementary material for: A cortical hierarchy of localized and distributed processes revealed via dissociation of task activations, connectivity changes, and intrinsic timescales
Source: Neuroimage. Author manuscript; Available in PMC 2021 Jan 3. (PMC7779074; doi:10.1016/j.neuroimage.2020.117141)
Supplement: 1 [file NIHMS1657519-supplement-1.pdf]

Supplementary Figures

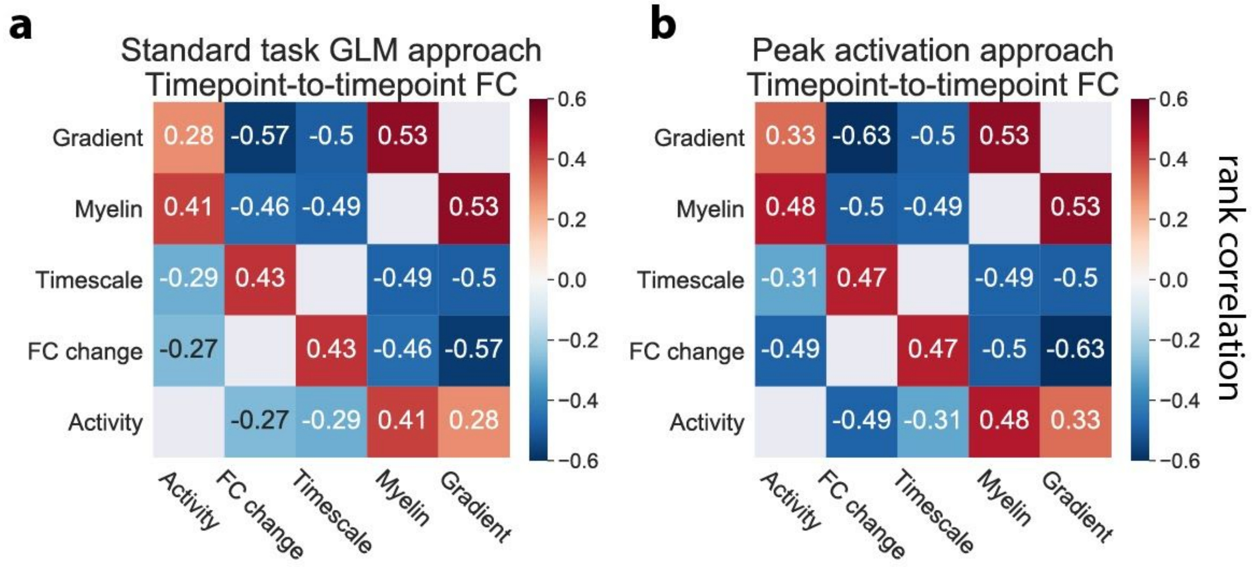

Supplementary Figure 1. Summary of positive and negative associations (on the replication cohort) between the resting-state principal gradient, task activations, task FC change, intrinsic timescales, and myelin content using the standard task GLM and the peak (block) activation approaches. a) The standard task GLM approach for the replication cohort. We use standard task GLM modeling to estimate activation coefficients for each brain region, and FIR task modeling to remove the mean task-evoked response prior to computing task FC. **b)** The peak activation approach for replication. We estimate the peak activation magnitude at each block (across all blocks) without task regression. Task activations are estimated by averaging peak magnitudes across all blocks for each brain region. Task FC estimates are obtained by correlating block-to-block variance (using peak magnitudes) between all pairs of brain regions. Positive and negative association strengths are typically stronger using the peak activation approach. All correlations were found to be statistically significant using an FDR-corrected p-value of $p<0.01$. All p-values were estimated using a spatial autocorrelation-preserving permutation test to generate random surrogate brain maps.

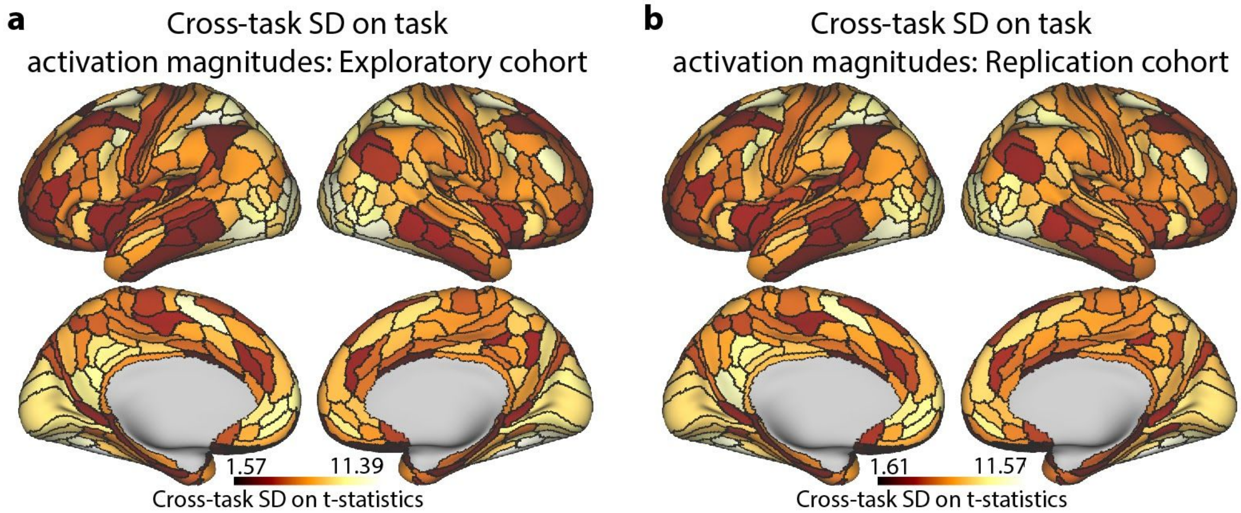

Supplementary Figure 2. The standard deviation (across tasks) of task activation magnitudes at each parcel. a) Cross-task SD (on 24 task conditions) on the task activation magnitudes (absolute value of t-statistic) for the exploratory cohort. **b)** Same as **a**, but for the replication cohort.

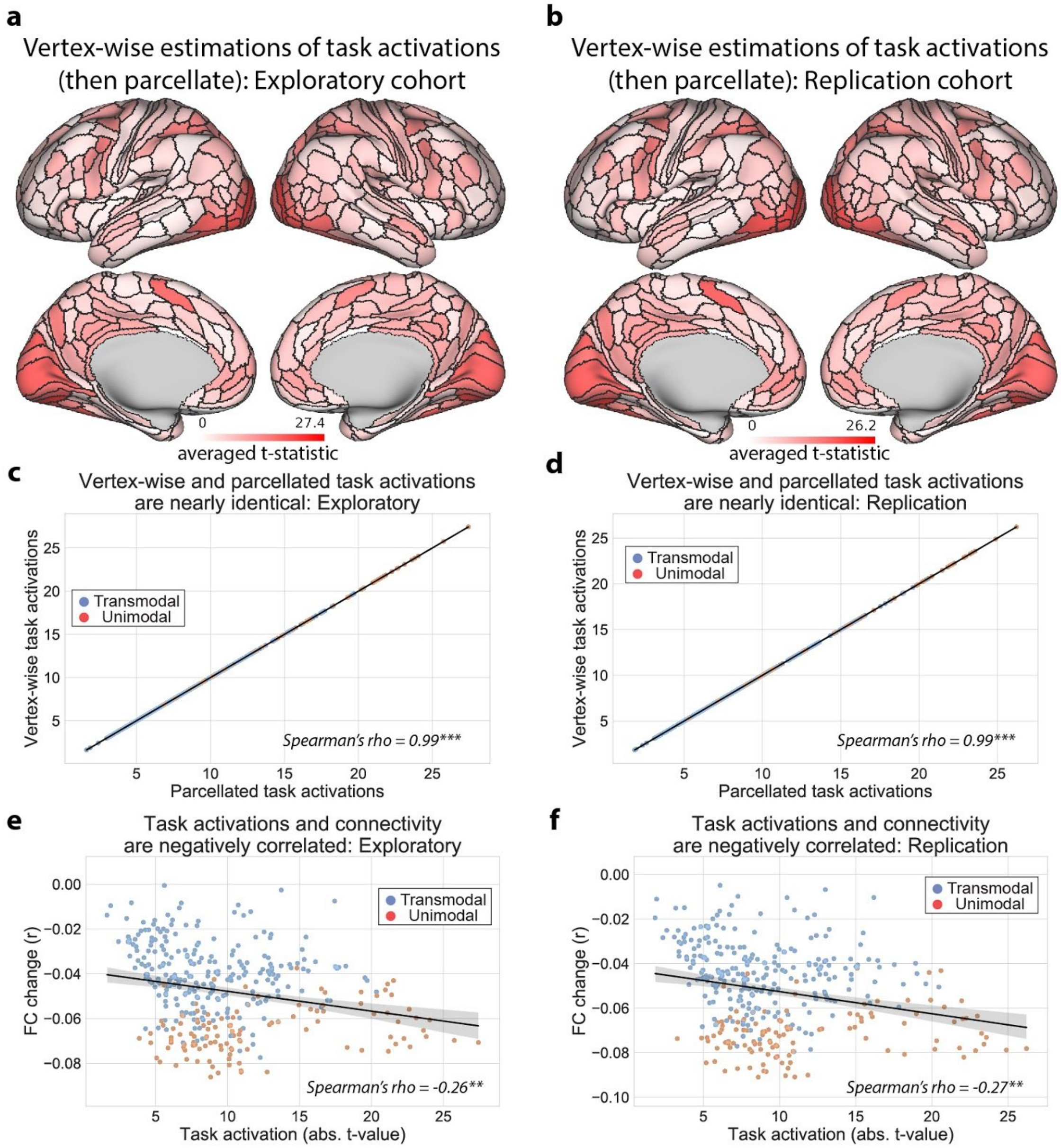

Supplementary Figure 3. Comparison of using vertex-wise task GLM estimates versus parcellated task GLM estimates for task activation estimation. a,b) We estimated the task activation maps by first estimating the task GLM coefficients for each vertex, and then parcellating the data (i.e., estimate-then-parcellate) for both the exploratory and replication cohorts. **c,d)** We compared the task activation maps using the estimate-then-parcellate approach versus the parcellate-then-estimate (i.e., parcellate data and then perform task activation estimation). Overall, we found that the approaches yielded virtually identical task activation estimates. We note, however, that no nonlinear preprocessing steps (e.g., z-scoring of time series) were performed, thus making it unlikely that any differences would be observed. **e,f)** We performed the same analysis as in Figure 2, where we compared the task activation estimates using the estimate-then-parcellate approach with the FC change estimates. Again, we found virtually identical results. (*** = p<0.001, ** = p<0.01, * = p<0.05)

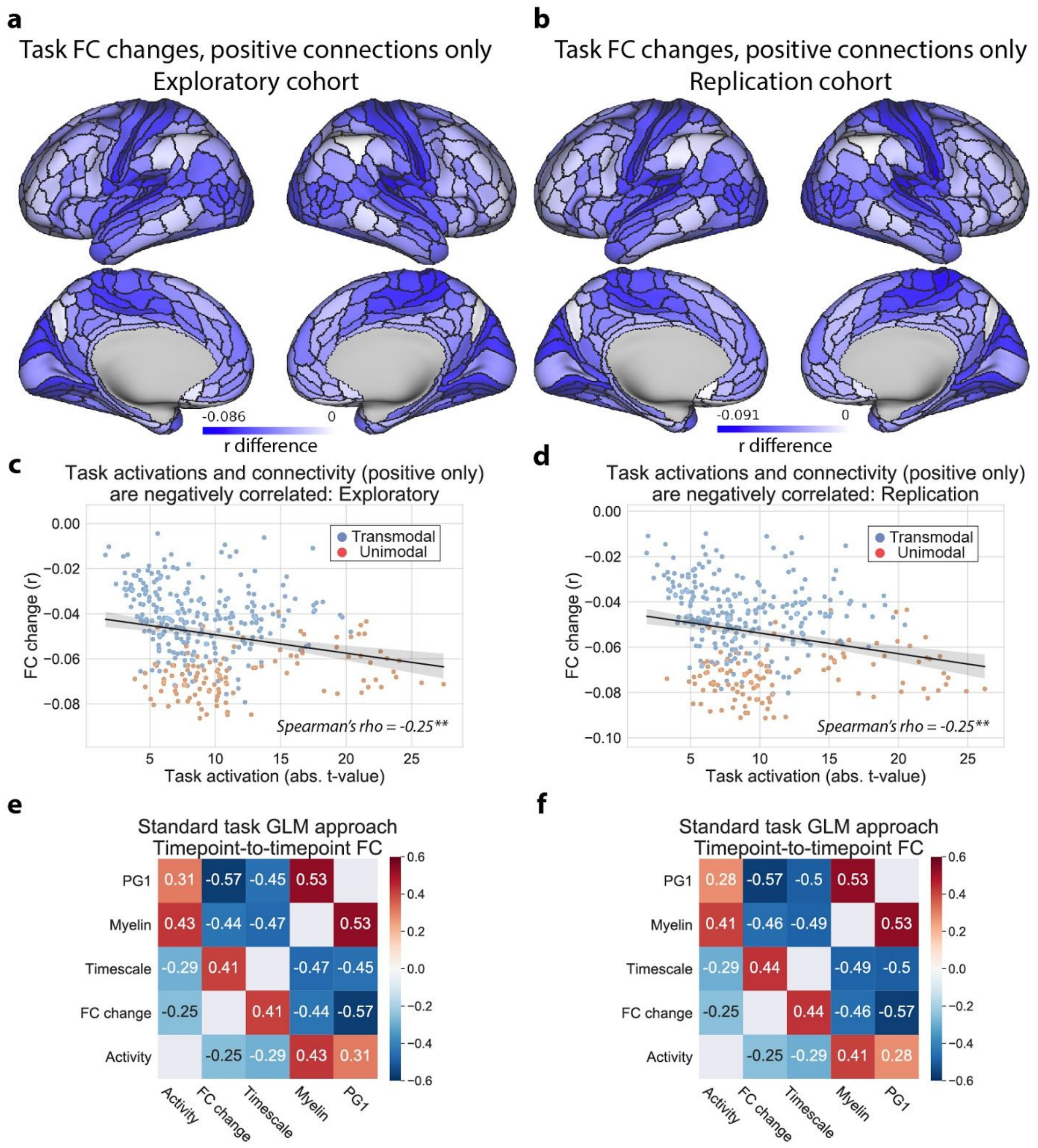

Supplementary Figure 4. Comparison of results using thresholded (positive only) FC matrices. We computed FC change values (i.e., task versus rest) on thresholded resting-state and task-state FC matrices, removing any negative FC estimates. (This was applied on each rest- and task-state FC matrix independently.) We found virtually identical results, corroborating our findings that task-state FC reductions are most prominent in sensorimotor areas, and reductions follow a hierarchical gradient. **a,b)** The average task-state FC change for every brain parcel, for the exploratory and replication cohorts. **c,d)** The correlation across parcels between task-state FC change and task-evoked activation magnitudes. **e,f)** The summary correlation matrix, demonstrating that the associations between task-state FC changes and task activation, intrinsic timescale, myelin, and PG1 maps are virtually unchanged when using positive FC values only. (Note that correlations between variables, such as myelin and timescale, will be unchanged from Figure 5, since the only changed variable here was the FC change map.)
